# Genetic Signals of Demographic Expansion in Downy Woodpecker (*Picoides pubescens*) after the Last North American Glacial Maximum

**DOI:** 10.1371/journal.pone.0040412

**Published:** 2012-07-09

**Authors:** Paulo C. Pulgarín-R, Theresa M. Burg

**Affiliations:** Department of Biological Sciences, University of Lethbridge, Alberta, Canada; University of Massachusetts, United States of America

## Abstract

The glacial cycles of the Pleistocene have been recognized as important, large-scale historical processes that strongly influenced the demographic patterns and genetic structure of many species. Here we present evidence of a postglacial expansion for the Downy Woodpecker (*Picoides pubescens*), a common member of the forest bird communities in North America with a continental distribution. DNA sequences from the mitochondrial tRNA-Lys, and ATPase 6 and 8 genes, and microsatellite data from seven variable loci were combined with a species distribution model (SDM) to infer possible historical scenarios for this species after the last glacial maximum. Analyses of Downy Woodpeckers from 23 geographic areas suggested little differentiation, shallow genealogical relationships, and limited population structure across the species’ range. Microsatellites, which have higher resolution and are able to detect recent differences, revealed two geographic groups where populations along the eastern edge of the Rocky Mountains (Montana, Utah, Colorado, and southern Alberta) were genetically isolated from the rest of the sampled populations. Mitochondrial DNA, an important marker to detect historical patterns, recovered only one group. However, populations in Idaho and southeast BC contained high haplotype diversity and, in general were characterized by the absence of the most common mtDNA haplotype. The SDM suggested several areas in the southern US as containing suitable Downy Woodpecker habitat during the LGM. The lack of considerable geographic structure and the starburst haplotype network, combined with several population genetic tests, suggest a scenario of demographic expansion during the last part of Pleistocene and early Holocene.

## Introduction

Continental North America has been a climatically and biologically dynamic place during the last two million years, especially during the last part of the Quaternary ice ages, 21,000 years ago when the polar ice sheets were at their maximum extent [1]. Two immense ice sheets (Cordilleran and Laurentide) covered most of Canada, and the northern part of the US at the LGM (last glacial maximum). Some areas north of the glacial maximum may have remained ice-free such as the Queen Charlotte Islands (Haida Gwaii), Alaska (Beringia) and parts of Newfoundland [2]. Most plants and animals probably survived in areas south of the ice sheets; quite different from present-day North America, where ∼20,000 species of vascular plants, more than 800 species of birds, and more than 640 species of mammals dominate a diverse and complex landscape [3]. The changing conditions following the melting of the ice sheets make this continent a natural laboratory to study patterns of colonization, geographic variation, adaptive radiation and extinction [4]–[10].

North America has been one of the main areas for studies on post-glacial expansion, and as such is an area where the impacts of the last glacial maximum (LGM) on organisms from a geological and biological perspective are best understood [7]. In North America the ice sheets were present as far as south as 40^o^N with the Pacific Northwest, Northeast coast, Southwest US and Beringia serving as the major refugia for a diverse array of organisms. Some examples of species that may have used these refugial areas include the Chestnut-backed Chickadee (*Poecile rufescens*), Steller’s (*Cyanocitta stelleri*) and Mexican Jays (*Aphelocoma ultramarina*) [11]–[13]; Northern Flying Squirrel (*Glaucomys sabrinus*), Red-backed Voles (*Clethrionomys gapperi*), American Marten (*Martes americana*); Spotted Salamander (*Ambystoma maculatum*) and Western Diamondback Rattlesnake (*Crotalux atrox*) [14,15 for a review on NW North America]. In contrast to Europe mountain ranges in North America run north-south and patterns of postglacial colonization from southern refugia differ between the two continents [1].

Despite the knowledge gained from multiple studies during the last two decades, the understanding of how different lineages and species responded in a spatio-temporal context to the retreat of the ice sheets following the LGM is still in its infancy [16]. How long did it take for organisms to colonize ice-free areas after the glaciers retreated? How much genetic structure remained after the colonization events? Even simple questions like these are not easy to answer for many taxa due to the analytical and methodological problems of inferring past events from current information, and the absence fossil data [17]–[20].

Studies on phylogeography and population genetics of Nearctic birds have uncovered an array of different trends [21], [22]. Multiple patterns are found in specialists and generalists, widespread and locally distributed species, and migrants and residents. For example, in some species, low intraspecific genetic diversity and absence of population genetic structure is one of the patterns found [21], [23], [24], [25] yet some of these species (*Junco hyemalis* and *J. phaeonotus*) have high morphological and ecological differentiation [25]. Other species have high genetic differentiation and geographic structure [26], [27]. Additionally it has been suggested a general east-west split in both genetic and plumage data reflects independent evolutionary histories and restricted gene flow [28]. The general patterns and the idiosyncratic nature of the genetic variation within each species challenge a simplistic view of the demographic and evolutionary history of the continental taxa [15], [21].

Patterns of genetic variation can be used to make predictions about the demographic and evolutionary history of an organism within the context of the LGM. One extreme are species with genetically structured populations displaying a gene genealogy with highly differentiated groups and collections of unique mitochondrial haplotypes or nuclear alleles restricted to specific areas, in addition to high nucleotide and haplotype diversities [12], [13]. This pattern is typical of colonization out of multiple refugia, by a large number of individuals and low levels of gene flow between populations. At the other extreme are species with reduced genetic structure and weakly differentiated populations. The expectations are shallow gene genealogies with one common haplotype and numerous, less common haplotypes that differ by a few mutational steps from the main one [29]. Populations also exhibit low levels of nucleotide diversity and no or very few private alleles. In most cases, these characteristics are typical of species that have undergone recent, rapid population expansions by a reduced number of individuals, usually from a single refugium [12], [24], [30]. Patterns can be species-specific as a consequence of different demographic or evolutionary histories [31].

**Figure 1:**
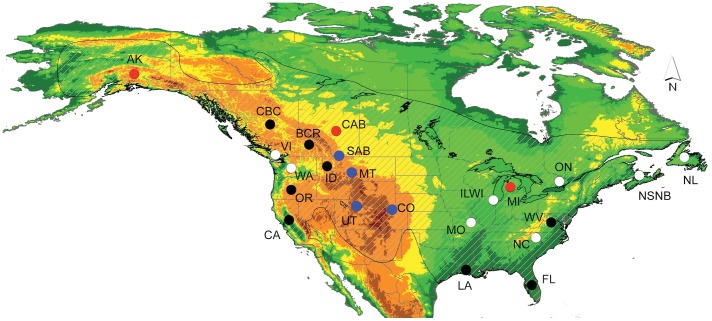
Distribution of the 23 sampling locations included this study. Abbreviations for localities are in Table 1. Coloured dots represent the distribution of the 15 populations with microsatellite information. Red dots correspond to populations with intermediate genotypes, and blue and white dots represent two well-differentiated groups in the STRUCTURE analysis (Fig. 3). Black dots are populations without microsatellite information. Crossed hatching indicates the general distribution of the species and colours increasing elevation from green to red [modified from 83].

Here we focused on the Downy Woodpecker (*Picoides pubescens*, Picidae) a small, sexually dimorphic woodpecker inhabiting a variety of habitats including coniferous, deciduous and mixed forest and tall shrubbery. It is a year-round resident ubiquitously distributed in North America from Alaska to Florida (Fig. 1) [32]. Some local movements between and within seasons have been reported, but it is not clear how often they occur and if they result in gene flow [33]. The Downy Woodpecker is represented by at least seven subspecies (*P. p. medianus, pubescens, glacialis, nelsoni, leucurus, gairdnelli* and *turati*) differing mostly in characteristics such as body mass and plumage coloration. Larger birds inhabit northern latitudes and higher elevations, and smaller birds live further south and at lower elevations [32], [34]. In addition, birds in the east are whiter than the ones in the west (e.g. more white spots on the wings). The five western subspecies are difficult to differentiate even when using several morphological and plumage traits [35]. Other North American species, such as the Hairy Woodpecker (*Picoides villosus*), the Brown Creeper (*Certhia americana*) and the Dark-eyed Junco (*Junco hyemalis*) present some similarities, for instance, there are several subspecies, and geographic variation in terms of body size and natural history traits [25], [28], [36].

We studied geographic patterns of genetic variation for the Downy Woodpecker (*Picoides pubescens*) using a series of population genetic statistics derived from mitochondrial DNA sequences and microsatellite data and created for the first time a species distribution model (SDM) at 21 kya. Preliminary evidence suggests that the Downy Woodpeckers were different than today’s in terms of population size and distribution during the late Pleistocene [21]. Ball & Avise [23] who used mtDNA RFLP data from 51 individuals mainly from the eastern US, found no genetic structure in the Downy Woodpecker. However, no study has attempted to examine a post-glacial expansion hypothesis using more rapidly-evolving molecular markers and a more complete geographic sampling for the Downy Woodpecker. Based on plumage and body size diversity, and reported “sedentary” behaviour [32], one might predict that in different areas of North America the Downy Woodpeckers have remained isolated. Previous genetic data [23] suggest a starburst pattern and little genetic differentiation, but had limited samples from glaciated or western regions. We predict that current Downy Woodpecker populations in previously glaciated areas are characterized by genetic signatures typical of species that have undergone rapid population expansion and small effective population size. Rapidly evolving markers such as microsatellites are better able to detect recent reductions in gene flow [37]. Given the morphological variation seen in the Downy Woodpecker, we predict nuclear loci will show reduced gene flow throughout parts of the range.

## Results

### Genetic Diversity

Fifty nine variable sites were found in an 850 base pair fragment of the mitochondrial genome for 263 individuals. No insertions or deletions were detected. Global nucleotide diversity (π = 0.00097) was low while haplotype (*H_d_* = 0.5839) diversity was moderate. Population π ranged from 0.0002–0.0031 and *H_d_* from 0.182 to 1.000 (Table 1). Among the 55 haplotypes, none differed by more than six nucleotide substitutions, and all the populations except Revelstoke, BC (BCR) shared a general widespread haplotype (Hap1). In general, Alaska, Washington, Newfoundland and southern Alberta were the least diverse regions in terms of *H_d_* and π, and Missouri, Idaho, Revelstoke (BC), and Louisiana exhibited the highest diversity for the same two genetic indices (Tables 1).

**Table 1:** Genetic diversity statistics for mtDNA and microsatellite markers across populations and loci.

Mitochondrial DNA						Microsatellites				
	Population	n	# hap	*H_d_*	π	n	# alleles	AR	*Ho*	*He*
AK	Alaska	11	2	0.18	0.0002	11	3.29	2.84	0.458	0.491
VI	Vancouver Island	–	–	–	–	11	4.71	–	0.685	0.620
WA	Washington	13	3	0.30	0.0004	14	5.14	3.87	0.699	0.612
OR	Oregon	8	4	0.64	0.0009	–	–	–	–	–
BCR	BC Revelstoke	9	5	0.86	0.0028	–	–	–	–	–
CBC	central BC	7	4	0.71	0.0010	–	–	–	–	–
CAB	central Alberta	19	5	0.46	0.0010	19	6.00	3.75	0.644	0.587
SAB	southern Alberta	13	3	0.30	0.0004	13	5.00	3.61	0.638	0.576
MT	Montana	21	5	0.73	0.0013	19	5.57	3.89	0.653	0.612
ID	Idaho	6	6	1.00	0.0031	–	–	–	–	–
UT	Utah	13	3	0.41	0.0005	16	4.57	3.21	0.626	0.539
CO	Colorado	19	6	0.54	0.0007	23	5.29	3.65	0.645	0.584
CA	California	7	3	0.67	0.0008	–	–	–	–	–
ILWI	Illinois/Wisconsin	18	9	0.76	0.0012	20	6.00	3.8	0.625	0.596
ON	Ontario	–	–	–	–	15	3.57	–	0.529	0.507
MI	Michigan	19	8	0.61	0.0010	19	5.71	3.66	0.616	0.583
NSNB	Nova Scotia/New Brunswick	15	5	0.48	0.0008	32	6.14	3.88	0.687	0.626
NL	Newfoundland	18	4	0.31	0.0004	16	4.71	3.41	0.613	0.568
WV	Western Virginia	5	2	0.40	0.0005	–	–	–	–	–
MO	Missouri	14	7	0.85	0.0018	15	5.71	3.81	0.671	0.580
NC	North Carolina	10	3	0.38	0.0005	14	4.71	3.69	0.647	0.598
LA	Louisiana	7	3	0.86	0.0014	–	–	–	–	–
FL	Florida	10	3	0.38	0.0005	–	–	–	–	–

Sample size (n), number of haplotypes (# hap), nucleotide (π) and haplotype (*H_d_*) diversities; average number of alleles (# alleles), allelic richness (AR), and observed (*Ho*) and expected (*He*) heterozygosities. Missing data are indicated by “–”.

* For microsatellites only populations with >10 individuals were used. For mitochondrial DNA, sequences from Ontario and from Vancouver Island (except one) were not available for population comparisons. One individual from Vancouver Island was included in the haplotype network analyses but excluded here.

MICRO-CHECKER showed no evidence of scoring errors due to allele dropout or stutter for any of the loci. The only evidence for null alleles arising from an excess of homozygotes was at DlU5. Both Illinois-Wisconsin (DMD9, *P* = 0.0217) and Vancouver Island (DMD118, *P* = 0.0360) deviated from Hardy-Weinberg equilibrium, but neither was significant after correction for multiple tests. No evidence of linkage disequilibrium was found among loci as indicated by Fisher’s exact test (*P* values ranged from 0.121 to 1.000).

The seven variable microsatellite markers had moderate levels of genetic variation among loci and across populations. The number of alleles per locus (and average number) ranged from 4 (2.7±0.159) in DMD9 to 17 (9.4±0.608) in DMD118. On average, Alaska had the lowest number of alleles (3.3±0.71), while Nova Scotia – New Brunswick had the highest (6.1±1.08) (Table 1). Microsatellite genetic diversity, measured indirectly using *H_e_*, was relatively uniform across the range with no association with latitude (*r^2^* = 0.1325, *P* = 0.1703) or longitude (*r^2^* = 0.0422, *P* = 0.4557).

### Population Structure

Mitochondrial-based pairwise *F*_ST_ comparisons across 21 sampling locations revealed minor differences among most pairs of populations (Table 2). Most of the significant differences were comparisons involving Revelstoke (14/20), and Idaho (8/20); however, a number of comparisons for Missouri (6/20), Montana (5/20), central Alberta (4/20) and Michigan (3/20) were also significant. The most common haplotype, Hap1, was absent from Revelstoke and found in a single bird in Idaho (n = 6) (Fig. 4). Plotting the genetic similarities (*F*_ST/_(1-*F*_ST_)) as a function of the geographic distance among populations revealed no significant relationship (*r^2^* = 0.0091, *P* = 0.220).

**Table 2:** Pairwise mtDNA (above) and microsatellites (below) *F*_ST_ values.

	AK	VI	WA	OR	BCR	CBC	CAB	SAB	MT	ID	UT	CO	CA	IL/WI	ON	MI	NS/NB	NL	WV	MI	NC	LA	FL
AK		0.080	**0.062**	–	–	–	**0.052**	0.047	**0.047**	–	**0.086**	**0.071**	–	0.039	0.030	0.036	**0.055**	**0.060**	–	0.051	0.024	–	–
VI	–		0.002	–	–	–	0.034	0.028	0.037	–	**0.074**	**0.051**	–	0.019	0.018	**0.046**	0.031	0.028	–	0.008	0.021	–	–
WA	−0.004	–		–	–	–	0.017	0.030	0.012	–	**0.072**	**0.042**	–	0.010	0.024	0.007	0.007	0.019	–	0.013	−0.007	–	–
OR	0.024	–	−0.017		–	–	–	–	–	–	–	–	–	–	–	–	–	–	–	–	–	–	–
BCR	**0.186**	–	**0.190**	0.103		–	–	–	–	–	–	–	–	–	–	–	–	–	–	–	–	–	–
CBC	0.041	–	0.037	0.001	0.129		–	–	–	–	–	–	–	–	–	–	–	–	–	–	–	–	–
CAB	−0.002	–	0.009	0.011	**0.207**	0.016		0.002	0.004	–	**0.030**	0.004	–	−0.001	0.023	0.003	0.015	0.008	–	−0.004	0.006	–	–
SAB	−0.004	–	−0.040	−0.017	**0.190**	0.037	0.009		0.020	–	0.043	0.015	–	0.016	0.038	0.019	0.020	0.014	–	0.000	0.022	–	–
MT	0.043	–	0.027	0.006	**0.160**	−0.010	**0.065**	0.001		–	0.023	−0.007	–	0.006	0.016	0.002	**0.030**	**0.038**	–	0.014	0.000	–	–
ID	0.121	–	**0.156**	0.061	0.050	0.043	**0.155**	0.156	0.093		–	–	–	–	–	–	–	–	–	–	–	–	–
UT	0.033	–	0.033	0.003	**0.214**	0.046	0.029	0.033	0.064	**0.166**		0.004	–	**0.036**	0.059	0.019	**0.073**	**0.081**	–	**0.064**	**0.053**	–	–
CO	−0.006	–	−0.013	−0.046	**0.208**	0.003	0.024	−0.013	0.050	0.166	0.000		–	0.017	0.028	0.016	0.049	0.050	–	0.028	0.029	–	–
CA	0.177	–	0.119	0.093	0.160	0.083	0.121	0.171	0.121	0.100	0.162	0.144		–	–	–	–	–	–	–	–	–	–
ILWI	−0.012	–	−0.001	−0.002	**0.186**	0.001	0.014	−0.001	**0.056**	**0.132**	0.017	0.016	0.097		0.000	0.002	0.014	0.011	–	0.005	−0.002	–	–
ON	–	–	–	–	–	–	–	–	–	–	–	–	–	–		0.015	0.015	0.021	–	0.011	0.008	–	–
MI	−0.039	–	−0.011	−0.004	**0.187**	0.001	0.014	−0.011	**0.054**	0.125	−0.013	0.008	0.108	0.007	–		0.009	0.030	–	0.022	0.004	–	–
NSNB	−0.013	–	−0.004	0.004	**0.199**	0.010	0.005	−0.004	0.053	**0.147**	0.020	−0.001	0.124	0.006	–	−0.011		0.004	–	0.007	0.002	–	–
NL	−0.010	–	−0.001	0.032	**0.256**	0.048	0.018	−0.001	0.066	**0.214**	0.036	0.011	0.193	0.001	–	−0.001	0.004		–	−0.011	0.013	–	–
WV	0.050	–	0.018	−0.024	0.099	−0.022	−0.028	0.018	−0.000	0.031	0.024	−0.020	0.071	−0.045	–	−0.047	−0.032	0.014		–	–	–	–
MO	0.072	–	0.082	0.049	**0.172**	0.045	**0.087**	0.082	**0.104**	0.117	0.088	**0.093**	0.106	0.040	–	**0.077**	0.054	**0.103**	0.008		0.001	–	–
NC	0.004	–	0.003	0.008	**0.181**	0.018	0.002	0.003	0.040	**0.129**	0.029	0.001	0.135	−0.009	–	−0.016	−0.008	0.004	0.000	0.062		–	–
LA	0.047	–	0.047	0.004	0.124	0.000	0.029	0.047	0.049	0.060	0.053	0.020	0.074	0.013	–	−0.001	0.023	0.063	−0.028	0.049	0.024		–
FL	0.004	–	0.003	0.008	**0.181**	0.018	0.002	0.003	0.040	**0.129**	0.029	0.001	0.135	−0.009	–	−0.016	−0.008	0.004	0.000	0.062	0.000	0.024	

Values in bold are significant after the Benjamini-Hochberg correction for multiple tests. Abbreviations for localities are in Table 1 and Figure 1.

Microsatellite pairwise *F*_ST_ comparisons among 15 sampling locations ranged from −0.007 to 0.086 (Table 2). In general, comparisons involving each of Alaska (7/14), Utah (9/14), Colorado (3/14), Washington (3/14), Vancouver Island (3/14) and Montana (3/14) were significant suggesting restricted gene flow across the species’ range. The isolation by distance analysis found a trend, but no significant correlation between genetic and geographic distance (*r^2^* = 0.1103, *P* = 0.070).

**Figure 2:**
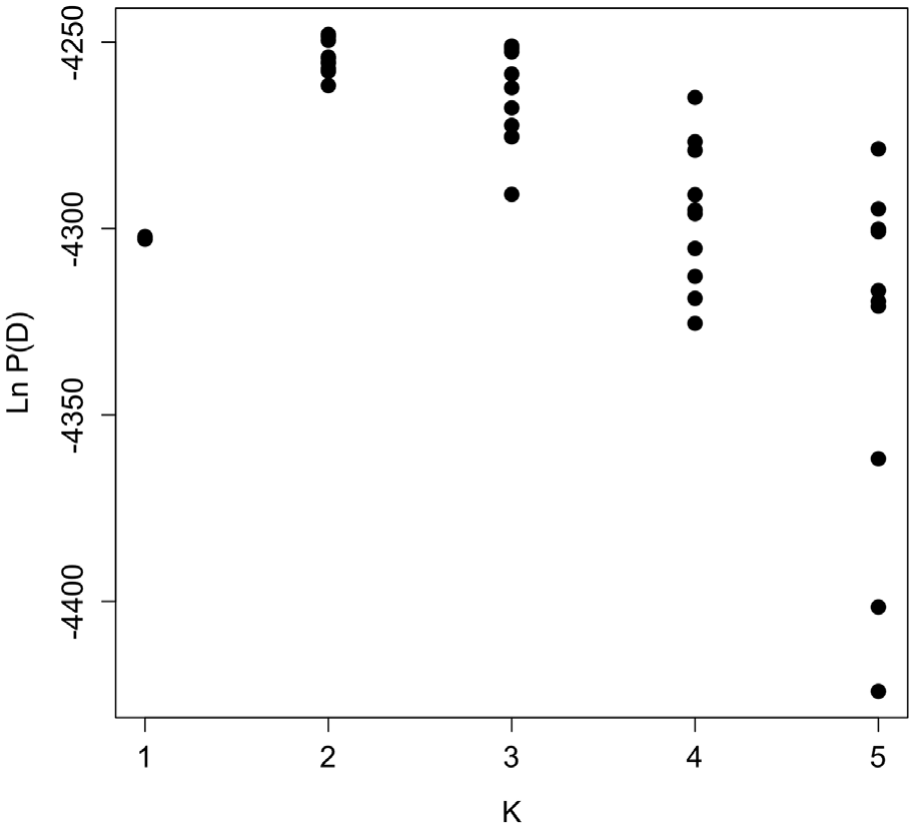
Posterior probability values from ten runs per given K (possible number of groups) in the program STRUCTURE. K = 2 is suggested by the lower mean value and using the Evanno et al. [**79**] method. The higher the ln P(X|K), the higher the probability of K being the most likely hierarchical group.

**Figure 3:**
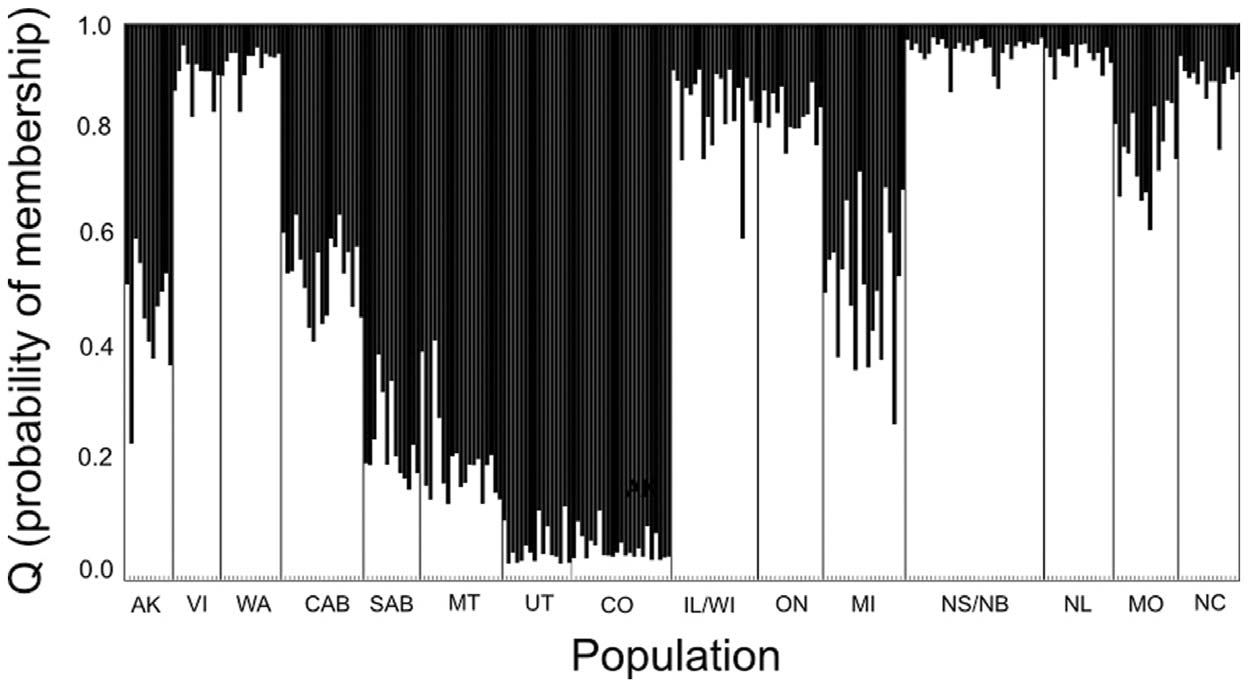
Barplot of assignment probabilities for 257 individuals from 15 populations. Geographic groups were arranged to display the admixture proportions (Q) for the sampled geographic areas. Each vertical bar (within the black lines) represents an individual from one population. Q values >0.6 are regarded as confident for population assignment of an individual to a single cluster.

The Bayesian clustering analysis estimated K = 2 as the most probable number of Downy Woodpecker subgroups using both ΔK method (Fig. 2) and Bayes factor (0.999 for K = 2). Based on assignment probabilities (coefficient of ancestry – *Q* >0.6) for each individual, one group includes Utah, Montana, Colorado and southern Alberta, and the second group includes most of the other populations. The exceptions to these two groups were Alaska, Michigan and central Alberta in which most individuals were equally assigned to both groups (Figs. 1 and 3).

### Phylogeographic and Demographic History

Statistical parsimony analysis of the 55 mitochondrial haplotypes produced a starburst pattern, a common, central haplotype different from most of the other haplotypes by one or two nucleotide differences, suggestive of a demographic expansion. Expansion times for the different populations suggest a time frame between 6,294 and 178,764 years, where the time of expansion for several areas is after the LGM (AK: 6,294; WA: 21,235; OR: 56,235; BCR: 178,764; CBC: 94,235; SAB: 21,235; MT: 66,882; ID: 176,882; UT: 30,823; CO: 44,823; CA: 110,823; ILWI: 73,882; MI: 54,647; NSNB: 36,764; NL: 22,823; WV: 33,294; MO: 98,882; NC: 28,529; LA: 85,588; FL: 28,823. See Table 1 for locality names). The hypothesis of constant population size was rejected by Fu’s F_s_ (−8.3204, *P*<0.0001). Likewise, Tajima’s D rejected a scenario of selective neutrality and population equilibrium (−2.65, *P*<0.0001). Fu and Li’s D* (−6.54, *P*<0.02) and F* (−5.77, *P*<0.02) were both negative and significant, supporting the results of Fu’s F_s_ and Tajima’s D. Nonetheless, a unimodal mismatch distribution signals that Downy Woodpecker populations have undergone periods of population growth (SSD = 0.00034, *P* = 0.75) (Fig. 5). This was also confirmed by the raggedness index, which failed to reject the null hypothesis of population expansion (r = 0.068, *P = *0.36).

**Figure 4:**
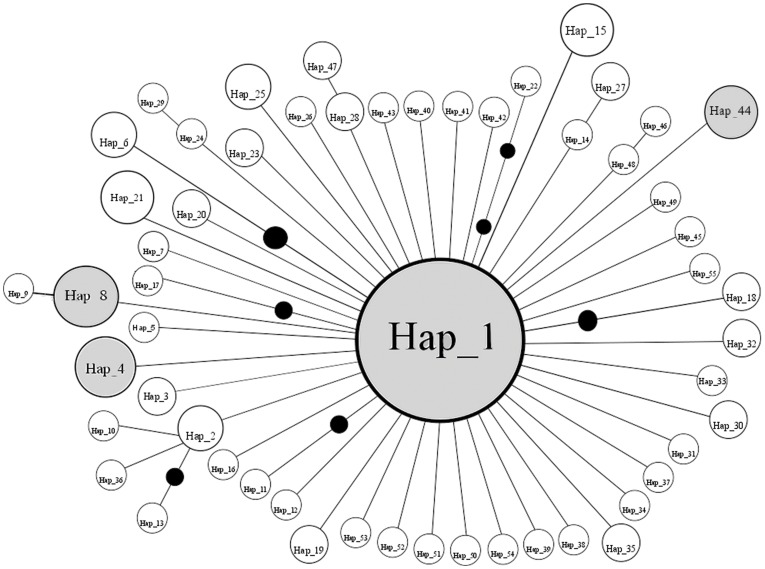
Statistical parsimony network of mtDNA haplotypes. The size of the circle indicates the number of birds with that haplotype. Hap1 was present in all the sampled populations with the exception of BCR (Revelstoke, BC). Black circles are inferred haplotypes.

The absence of any geographic pattern in the haplotype distribution in the TCS network suggests limited phylogeographic structure of breeding populations of the Downy Woodpecker in North America (Fig. 4). A single widespread haplotype (Hap1) was present in all the populations except Revelstoke, BC. Hap1 was the most frequent haplotype found in 169 individuals (64%), followed by Hap8 (9 ind. –3.4%), Hap4 (6 ind. –2.3%) and Hap44 (5 ind. –2%). The remaining 51 haplotypes were present in one to four individuals (Fig. 4 and Table 3).

**Figure 5:**
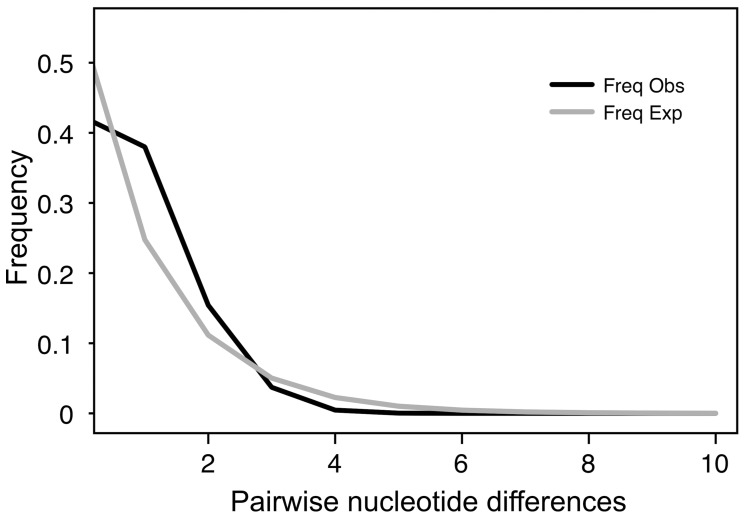
Mismatch distributions of pairwise nucleotide differences between mtDNA haplotypes revealed a unimodal pattern and no significant differences between the observed and expected frequencies (sum of squared deviation = 0.000344, *P* = 0.75). The black line is the observed frequency and gray line is the expected frequency.

**Table 3:** Geographic distribution of haplotypes.

	AK	VI	WA	OR	CA	BCR	CBC	CAB	SAB	MT	ID	UT	CO	ILWI	MI	NSNB	NL	WV	MO	NC	LA	FL	Total
**Haplotype**																							
**1**	10	1	11	5	4		4	14	11	10	1	10	13	9	12	11	15	4	5	8	3	8	169
**2**	1					2																	3
**3**							1						1										2
**4**							1			4	1												6
**5**							1																1
**6**						3																	3
**7**						1																	1
**8**			1	1		2			1	3			1										9
**9**						1																	1
**10**											1												1
**11**											1												1
**12**											1												1
**13**											1												1
**14**			1																				1
**15**				1								1	2										4
**16**				1																			1
**17**								1															1
**18**								2															2
**19**								1						1									2
**20**								1								1							2
**21**									1	3													4
**22**										1													1
**23**													1								1		2
**24**													1										1
**25**												2			1								3
**26**					1																		1
**27**					2																		2
**28**																1			1				2
**29**																1							1
**30**															1	1							2
**31**																	1						1
**32**														1			1						2
**33**																	1						1
**34**															1								1
**35**															1						1		2
**36**															1								1
**37**															1								1
**38**															1								1
**39**														1									1
**40**														1									1
**41**														1									1
**42**														1									1
**43**														1									1
**44**														2					3				5
**45**																			1				1
**46**																			1				1
**47**																			2				2
**48**																			1				1
**49**																				1			1
**50**																				1			1
**51**																		1					1
**52**																					1		1
**53**																					1		1
**54**																						1	1
**55**																						1	1
**Total # ind**	**11**	**1**	**13**	**8**	**7**	**9**	**7**	**19**	**13**	**21**	**6**	**13**	**19**	**18**	**19**	**15**	**18**	**5**	**14**	**10**	**7**	**10**	263

### Ecological Niche Modeling

The resulting models for the current and LGM 21 kya Downy Woodpecker distribution in North America were constructed based on 16,271 presence records used for testing and 5,423 for training. The predicted models for the current distribution and at 21 kya were statistically robust using guidelines suggested by [38]. The omission plot (not shown) displayed a good match between the predicted omission by the program algorithm and the training data (25% of locality records). Both the training data (5,423 records) and the test data (16,271 records) predicted similar models, as the area under the curve test - AUC = 0.942 (values can range from 0 to 1, and values less than 0.5 are not better than a random model).

**Figure 6:**
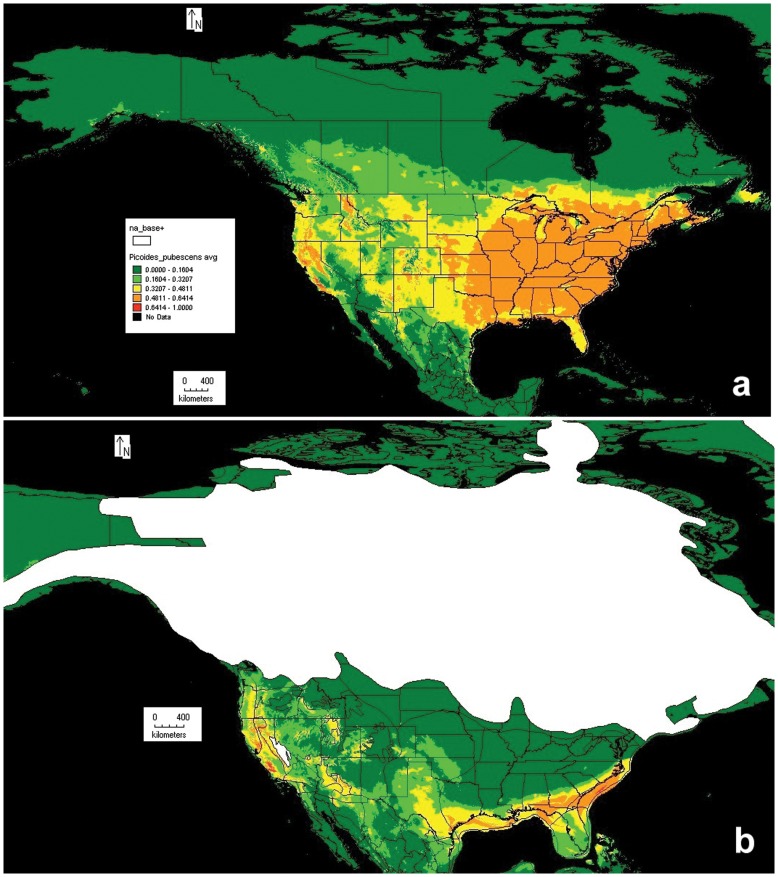
Current (a) and LGM (b) species distribution model of the Downy Woodpecker as modeled with 19 bioclimatic variables [**see 81**]. Warmer colors reflect areas with higher probabilities of occurrence. The distribution of the ice sheets (white) follows [39].

The predicted model for the current distribution suggests a strong correspondence with other sources of information related to the current species’ distribution (Fig. 6a). The predicted distribution during the LGM (21 kya) suggests two main areas (Fig. 6b). The largest area corresponds to the southeastern US from North Carolina west to Texas, with a proportion of the suitable habitat outside the current coastal line (sea level dropped up to 120 m at 21 kya [39]). The second main area corresponds to the Rockies with higher distribution probabilities in the east: Arizona, Utah, and Colorado, and west: California and Oregon. At the LGM these areas were covered with open boreal woodlands, semi-arid temperate woodlands or scrub, forest steppe, subalpine parkland, temperate steppe parkland and taiga [39]. Based on current habitat preferences [32], it seems that all of the above could have been potential places for the Downy Woodpecker to persist.

## Discussion

### Congruencies and Contrasts between mtDNA and Microsatellites

This study examined on rangewide patterns of genetic variation in the Downy Woodpecker in the context of postglacial expansion and colonization of North America and expands on previously published work [23]. Both mtDNA and microsatellite data showed limited genetic structure across sampled locations, with most geographic groups characterized by a wide range of haplotype and nucleotide diversities, and similar expected heterozygosity. The presence of a common, widespread haplotype and a unimodal mismatch distribution suggest a recent common history for most continental North American populations. The exceptions to this are Downy Woodpeckers in Revelstoke (BC) and Idaho, as discussed below. Microsatellite and mtDNA data suggest Alaska has the lowest genetic diversity (Table 1) compared with the other sampled populations and support the idea of a recent founder event or a bottleneck. In comparison, Washington and southern Alberta have low nucleotide and haplotype diversities in contrast to high nuclear variation. Both Washington and southern Alberta sampling sites are adjacent to Revelstoke and Idaho (Fig. 1) suggesting that geographic barriers such as the Rocky Mountains or different colonization routes could explain the current patterns of genetic variation in nearby areas. While sample sizes from the Revelstoke and Idaho populations were too small to obtain accurate allele frequency estimates, the mtDNA haplotypes from these two populations show uncharacteristically high haplotype variation, unique haplotypes and low frequency of Hap1.

Patterns in genetic diversity indices for mtDNA and microsatellites have been shown to be similar, but can also vary in the same species [8]. This may reflect not only the intrinsic mutational processes, but also the effective population size associated with nuclear and mitochondrial genomes or selection [8], [40]. In addition, it may reflect more contemporary processes such as restricted gene flow between populations or male-biased dispersal, a pattern that can be detected by the microsatellites but not mtDNA [41].

### Population Structure

*F*_ST_ based differences suggest partially incongruent patterns between mtDNA and microsatellite data, which is expected under equilibrium due to different effective population sizes and other factors, such as higher mutation rates in microsatellites than mtDNA [8], [42]. For instance, southern Alberta, Montana, Utah and Colorado were differentiated from other populations with the microsatellite analyses (Fig. 3), but, with the exception of Montana, not using mtDNA (Table 2). Most of the genetic variation (>90% in AMOVAs, not shown) is contained within populations and IBD analyses for both datasets suggest gene flow may be restricted by physical distance though support is weak (mtDNA: *r^2^* = 0.0091, *P* = 0.220, microsatellites: *r^2^* = 0.1103, *P* = 0.070). Therefore, over a large geographic scale, the effects of genetic drift may be stronger than gene flow explaining why patterns of significant *F*_ST_ values do not necessarily correspond to geographically clustered populations. A similar pattern albeit at a different geographic scale is found in the Greater Prairie-Chicken (*Tympanuchus cupido*) [43].

Three sets of populations: Alaska, populations east of the Rocky Mountains and populations in the Intermountain West (Revelstoke BC and Idaho) show evidence of restricted gene flow. The patterns for the first two sets of populations are only evident with microsatellite markers. In the case of Alaska allele frequency differences may be the result of a founder event as evident from the low mtDNA variation (H_d_ = 0.18); however, tests for recent bottlenecks were not significant (*P* = 0.62). In contrast, gene flow in the eastern Rockies (MT, CO and UT) may be restricted by a number of factors including habitat and landscape barriers [see 44,45]. Restricted gene flow may also be evident in the Intermountain West with nuclear loci, however, additional samples will need to be collected to test this. Nevertheless, Intermountain West populations harbour uncharacteristically high mtDNA variation that we address below.

As gene flow is not significantly impeded by geographic distance in the Downy Woodpecker (as suggested by genetic analyses and band recovery data), it is possible that populations are not reproductively isolated, and individuals are moving between distant geographic areas. However, we cannot claim there is or has been continuous gene flow since some populations (e.g. Illinois-Wisconsin or Missouri) have higher haplotype (*H_d_*) diversities and allelic richness (AR) than others (Table 1) and restricted gene flow accessed by *F*_ST_ can also indicates potential contact zones. Browning [33] found that of the 113 band recoveries of Downy Woodpeckers, 94% were hatch year (HY) and after hatch year (AHY) females moving between areas between different seasons (on average 218 km). Three of the recoveries were 583 km (winter to summer), 591 km (fall to winter) and 1080 km (summer to winter) away from the original banding locations. This potential scenario of birds (>90% females) moving seasonally, even at low frequencies, could in theory genetically homogenize the populations [46], [47]. If females’ movements are higher in some geographic areas, this would have had a strong influence on the genetic architecture of different populations for both microsatellites and mtDNA. Although not fully understood, it seems that Downy Woodpeckers (mostly females) disperse during the breeding season searching for food and empty territories [48]. It is not known if there is differential dispersal between the east and west due to bird density, resources or physical barriers [32].

### Demographic History

One of the most relevant findings of this study is related with the genetic distinctiveness of the BC Revelstoke and Idaho populations. Despite the small sample size and the lack of microsatellite data, this highlights the importance of southern BC and Idaho as a potential refugium for animals and plants [11], [49], [50], [51]. Although our data (genetics and SDM) do not allow us to conclusively state a refugia in the northern Rocky mountain area (such as Clearwater), it is congruent with a widespread pattern where populations southern of the ice sheets are genetically more diverse (see also Missouri and Louisiana populations, Table 1) than their northern counterparts [28], [52]. It is also intriguing that southern Alberta, geographically very close to Idaho and BC Revelstoke, had one of the lowest genetic diversity measures at both mtDNA mitochondrial and nuclear loci. Consequently, one possible explanation for the low genetic diversity in the eastern slope of the Rocky Mountains (and the forested areas in southern Alberta plains) and other areas such as Washington and Newfoundland is that they were recently colonized either from the southern populations or refugial areas in the northern Rockies [51], [52].

Our estimates of time since expansion (using a standard mutation rate of 2%/Ma) suggest that current Downy Woodpecker populations expanded from 178,764 (BC Revelstoke) years to 6,294 years (Alaska). Several populations such as Washington and southern Alberta expanded around the 21 kya period whilst other (California, Missouri and Louisiana) predates the LGM boundary. Although these estimates can be a reference to understand the demographic history, the putative times should not be taken as conclusive as they are highly dependent on the mutation rates.

In much the same way, the genetic patterns depicted here are suggestive of at least three explanations for the Pleistocene demographic history of this species. One is the rapid population expansion typified by starburst as seen in the mtDNA network (Fig. 4) and supported with the mismatch analysis (Fig. 5). Second, a new advantageous allele might have spread throughout the population and, as the mitochondrial genes are linked, a selective sweep at one mitochondrial locus may have reduced mtDNA genetic variation at “neutral” loci [53], [54]. We cannot rule out background selection based on Fu and Li’s D* and F*, however, selection may not have acted on ATPase genes themselves. As the mitochondrial genome is composed of a number of coding loci, selection at any locus would reduce variation at the ATPase loci. The third possibility is a population bottleneck. Reduction in habitat and resources during an interglacial or glacial might have decreased the genetic variation [47]. Higher nuclear variation does not rule out any of the three possibilities.

Several independent lines of evidence support a postglacial expansion by the Downy Woodpecker during the last 21 kya. First, a star-like haplotype network characterized by a predominant haplotype and accompanied by a collection of low frequency peripheral haplotypes differing by one or two mutational steps from the main haplotype [12], [23], [29], [55]. Second, is the absence of divergent haplotypes between geographic groups (even with reduced structure) as shown in the current study [24], [56]. Third, a unimodal mismatch distribution not different from a model of sudden expansion, which suggests an historical increase in effective population size [31]. More support comes from the neutrality test, where Fu’s F_s_ and Tajima’s D rejected the null hypothesis of population stability.

Finally, the species distribution model for the Downy Woodpecker at 21 kya suggests two distinct refugial areas, one in the southeastern US and the second in the west from Arizona to Oregon. Assuming that this distribution model represents a realistic scenario, it strongly favors a limited number of southern refugia. The predicted LGM habitat does not support any additional areas north of the ice sheets, such as Beringia, for Downy Woodpeckers. From a genetic point of view, both mtDNA and microsatellite data, Alaska has the lowest genetic diversity of all the populations (and lacks unique genetic variants), suggesting that it was recently colonized from other populations. Finally, mtDNA data do not support surviving lineages originating from multiple refugia; however the nuclear data do support the presence of at least two genetically distinct groups. It is possible that a reduction in gene flow detected in the microsatellite data arose after the LGM.

Our results show limited phylogeographic structure in the Downy Woodpecker supporting a previous mtDNA RFLP study [23]. Unlike Ball & Avise [23] we found limited, but significant structure along the eastern Rocky Mountains in Montana, Colorado, Utah and southern Alberta. Another novel finding of this study is that Alaska (mtDNA and microsatellite data) has the lowest genetic diversity of all the populations (and lacks unique genetic variants), suggesting that it was recently colonized from other populations. The genetic affinities of non-sampled populations in the Midwest is unknown, however, based on the current genetic patterns it is unlikely that the inclusion of samples from this area will change the general patterns depicted here.

## Materials and Methods

### Sampling and DNA Extraction

Individuals were sampled through: fieldwork (blood samples), toe-pads from museum birds, or tissue loans from different institutions. A total of 262 birds were used for mtDNA and 257 for microsatellite analysis (see Table 1) with differences in sample sizes explained below. All animals captured in the field for sampling were properly treated and released in the field. No birds died during the procedures. Blood samples were collected following animal welfare protocol (#0624) approved by the University of Lethbridge animal welfare committee using guidelines set by the Canadian Council on Animal Care (CCAC). Most samples were collected during late spring-summer and include individuals from across the majority of the species’ range (Fig. 1) and all seven subspecies recognized by the American Ornithologists’ Union (AOU) [32].

Total genomic DNA was extracted from muscle, blood, feather shaft and toe-pads from bird skins using a modified Chelex extraction [40]. Ten microlitres of blood-ethanol mix, or the equivalent in muscle or toe-pad, was incubated at 50°C for 30 min to allow the ethanol to evaporate. A 300 µL solution of DNA extraction buffer (0.1 M Tris-HCl pH 8.0, 0.05 M EDTA, 0.2 NaCl and 1% SDS) containing 5% w/v Chelex, 500 µg of proteinase K and 250 µg RNase was added, and the sample was incubated at 50°C overnight on a rotating wheel. The sample was vortexed, centrifuged at 10,000 rpm for 60 s, and 200–250 µl supernatant of transferred to 300 µl 5% w/v Chelex in low TE buffer (10 mM Tris-HCl pH 8.0, 0.1 mM EDTA).

### Mitochondrial DNA Amplification and Sequencing

Partial tRNA-Lys, ATPase 6 and 8 genes were sequenced for 263 individuals (GenBank accession numbers: JX094511-JX094773). We were able to amplify the targeted regions for 262 individuals; however, some museum specimens could not be amplified (e.g. Vancouver Island or Ontario, Table 1). DNA amplification was carried out in 25 µl reactions under the following conditions: ∼100 ng DNA, 250 µM dNTP, 2.5 mM MgCl_2_, 15 pmol of each primer (L8929 - COII 5′- GGMCARTGCTCAGAAATCTGYGG -3′ and H9855– ATP6 5′-ACGTAGGCTTGGATTAKGCTACWGC -3′) [57], PCR buffer, and 1 U of Taq polymerase. Reactions consisted of: one cycle of 120 s at 94°C, 45 s at 58°C, 60 s at 72°C; 37 cycles of 30 s at 94°C, 45 s at 58°C, 60 s at 72°C; and one cycle of 300 s at 72°C and 20 s at 4°C. Five microlitres of PCR product was incubated with 0.1 units exonuclease and 0.1 units of SAP for 15 min at 37°C followed by 15 min at 80°C to inactivate the enzymes. Sequencing consisted of a 10 µl reaction volume: 0.25 µl Big Dye (ver. 3.1), 3.5 µl 2.5X Big Dye buffer, 2.5 pmol of primer (L8929 COII), and approximately 100 ng of purified PCR product. The program consisted of one cycle of 120 s at 96°C; 25 cycles of 30 s at 96°C, 15 s at 50°C and 240 s at 60°C. PCR products were cleaned using a NaC_2_H_3_O_2_ precipitation. Samples were left to incubate at 4°C for 20 min and centrifuged at 13,000 rpm for 20 minutes, followed by two sequential rinses to remove residual salts: first with 100 µl cold 70% ethanol, spun at 13,000 rpm for 5 min, and second 35 µl cold 70% ethanol centrifuged at 13,000 rpm for 5 min. Ethanol was removed after each step. Samples were left to evaporate in the dark at room temperature for 60 min, re-suspended in 10 µl of hi-di formamide, and sequenced using an ABI3130 sequencer.

### Microsatellite Amplification and Scoring

Only populations with ten or more individuals were genotyped (Table 1). Samples were screened at 12 published microsatellite loci [58], [59], [60]. Five of the 12 were monomorphic (DlU1, DlU4, Ptri5, DMC112, and DMD7) and were dropped. A total of two hundred and fifty seven individuals were genotyped at seven variable microsatellite loci: DlU5 [58], DMA2, DMD9, DMC111, DMC115, DMD118 [59], and Ptri3 [60]. All ‘forward’ primers were modified with the addition of a M13 sequence on the 5′ end to allow for the incorporation of a fluorescently labelled M13 primer during PCR [13]. The forward primer of DMC115 (5′-TGTCAGAGATGGTTCATGGGTGCACT-3′) [59] and Ptri3 (5′-GCAAAAGCCAGTTCCTGTGCATGG-3′) [60] were modified to improve DNA amplification. All loci were amplified using a two-step procedure that consisted of: one cycle of 120 s at 94°C, 45 s at annealing temperature 1 (T_A1_), and 60 s at 72°C; seven cycles of 60 s at 94°C, 30 s at T_A2_, 45 s at 72°C. Annealing temperatures (T_A1_ and T_A2_, respectively) were 45–48°C for DMA2, DMD9, DMD118; 48–50°C for DMC111; 50–52°C for: DlU5, DMC115 and Ptri3. DNA amplification was carried out in 10 µl reactions under the following conditions: ∼200 ng DNA, 100 µM dNTP, 2.5 mM (for DMC111, DMD118, DMA2, DlU5, Ptri3) or 1.5 mM (DMD9, DMC115) MgCl_2_, 5 pmol of each F and R primer (and M13 primer), 1X PCR buffer, and 0.5 U of Taq polymerase. Amplification products were loaded on a Li-COR 4300 analyzer. DNA fragments were sized using Li-COR size standards and internal controls (three samples run on every load). Both authors checked microsatellite genotypes for every locus independently. We also developed a check plate containing multiple samples from each gel to confirm the original scores and check for shifts in allele sizes during scoring.

### Mitochondrial DNA Genetic Analyses

Sequences were aligned manually using MEGA version 4 [61]. Two measurements of DNA polymorphism, nucleotide (π) and haplotype (*H_d_*) diversity indices [62], were calculated for each population and all populations combined using DnaSP version 4.50.1 [63]. To understand how the genetic variation was partitioned among and within populations, Wright’s fixation index *F*_ST_ was calculated in Arlequin version 3.1 [64]. We tested for a positive association between genetic (*F*_ST_/(1-*F*_ST_)) and geographic distances with those expected under a stepping-stone model of population structure [65] using isolation by distance (IBD) analysis. A Mantel test was used to test significance with 500,000 permutations in GenAlEx version 6 [66].

Phylogeographic and demographic history of the Downy Woodpecker were studied using two complementary approaches. First, genealogical relationships among haplotypes were visualized using the software TCS version 1.21 [67]. This program produces a parsimony-based minimum-spanning network to display the number of possible connections (nucleotide differences) between haplotypes at a 95% confidence level. Second, we used classical population genetic statistics. Fu’s F_s_ is very powerful at detecting population expansions (increases in effective population size) by estimating departures from neutrality [68], [69]. Large, significant negative F_s_ values reject the null hypothesis of population stasis. F_s_ were calculated in DnaSP version 4.50.1 and significance was evaluated with 10,000 coalescent simulations. Similarly, Tajima’s D was used to test against selective neutrality and population equilibrium [70] where negative and significant values suggest either population expansion or purifying selection. Tajima’s D was calculated in Arlequin version 3.1 [64]. Additionally, Fu and Li’s D* and F* tests were calculated to determine if background selection explained the patterns of genetic diversity in this species. If Fu and Li’s statistics are significant and Fu’s F_s_ and Tajima’s D are not, then background selection is more likely creating the observed pattern [68].

We used mismatch distributions to help visualize signatures of demographic expansion for all the samples combined, and to test the null hypothesis of population growth [30], [31]. The simulated distribution of pairwise nucleotide differences (under the population growth model) was compared with the observed distribution using the sum of square deviations (SSD). Likewise, the raggedness index (r) was calculated to test if the frequency of pairwise nucleotide differences followed a smooth unimodal curve expected from a growing population by comparing the expected and observed (r). Small values of r suggest a good fit to the model. Both SSD and r were calculated in Arlequin version 3.1 [64].

We estimated the putative time of population expansion most of the populations from the statistic tau (τ). τ is equal to 2ut, where u equals 2 µk, μ is the mutation rate and k is the length of the sequence and t the time of expansion [31]. Tau (τ) values were calculated in Arlequin version 3.1 [64]. Population expansion times were estimated assuming a constant molecular clock and rates from 0.5, 1.6 and 2% (71 Pereira & Wajntal 2008) using the only tool http://www.uni-graz.at/zoowww/mismatchcalc/mmc1.php developed by [72].

### Microsatellite Genetic Analyses

MICRO-CHECKER [73] was used to detect errors in scoring and the presence of null alleles. Allele frequencies, allelic richness, observed (*Ho*) and expected heterozygosity (*He*) were calculated for every population and each locus using the package GenAlEx version 6 [66]. Linkage disequilibrium and Hardy-Weinberg equilibrium were assessed (using Fisher’s exact probability test) in the web version of GENEPOP version 4.0.10 [74]. The Markov chain parameter was set to 10,000 dememorizations, 1000 batches, and 10,000 iterations per batch for both analyses.

Genetic differentiation among sampled populations was evaluated using *F*_ST_ [75] implemented in GENETIX version 4.05 [76]. A total of 10,000 permutations was used to determine the significance of the *F*_ST_. For microsatellite analysis of population structure, *F*_ST_ is preferred over *R*_ST_ when the number of loci is less than 20 [77] as is the case with this study.

The Bayesian clustering program STRUCTURE version 2.3.3 [78], [79] was used to determine the number of possible clusters in our sample (K). K was set to a value from 1 to 16 during the initial exploratory runs (500,000 generations with a burn in of 100,000), and was then reduced to K = 1–5 for the final analyses based on the number of clusters detected in the initial runs. The final analysis was run for 1,000,000 generations with a burn in of 500,000, assuming population admixture (same alpha for the degree of admixture), correlated allele frequencies, and including *a priori* population information. Ten replicates were performed at each value of K for the initial and final runs. Average ln P(X|K) was plotted against K to visualize the number of possible clusters as suggested by the authors. Although STRUCTURE is widely used, the biological meaning of the number of groups (K) can be problematic to infer when there are few available loci or the populations are not strongly differentiated [79]. Likewise, because it is difficult to decide which K captures the major structure in the data due to similar ln P(X|K) values, two complementary methods were employed. One is the Bayes rule, where posterior probabilities for each K are compared in order to select the more meaningful K [78]. The second is the ΔK method [79], for which we used the on-line tool STRUCTURE Harvester (http://taylor0.biology.ucla.edu/struct_harvest/). ΔK eliminates the possibility of overestimating the potential number of populations or groups due to the fluctuating nature of probability ln P(X|K) as K increases. One limitation with the ΔK method is it cannot detect when K = 1 is the true number of clusters. Finally, isolation by distance (IBD) analysis was carried out for microsatellites in order to test the relationship between genetic and geographic distances.

### Ecological Niche Modeling (ENM)

To gain a better understanding of the possible areas where the Downy Woodpecker survived during the LGM, a distribution model was constructed using the maximum entropy method implemented in Maxent version 3.3.3e [38]. Maxent estimates maximum entropy distributions using climatic variables to predict non-negative probabilities on a target distribution using presence records [80]. The analysis is based on 19 bioclimatic variables from the WorldClim dataset, publically available and extensively used in ENM [14], [81]. For all analyses, we used the default convergence threshold and maximum number of iterations (500), using 25% of the localities for model training. Maxent produces a continuous probability value (0 to 1 using the logistic default output) as an indicator of the presence/suitability for the species based in the principle of maximum entropy [38]. Distribution modeling for the LGM was done using paleoclimatic data drawn from the Community Climate System Model [82].

Sets of 16,271 localities were used to generate current and historical distribution maps. All the records were downloaded from the Global Biodiversity Information Facility (GBIF) data portal (http://data.gbif.org), and included the localities used in the genetic analyses. Duplicate records were removed as part of the default setting in Maxent to avoid pseudo-replication. To test if the modeled distribution of the Downy Woodpecker during the LGM corresponds to a realistic model, we used two basic techniques implemented by the software. The first is the omission plot. If the data using for training deviates from the predicted omission line then the model is statistically weak. The second if the area under the curve (AUC), which test if the model predicted with the training data (25% of the total locality records), is similar to the testing data, and if both are not better than a random model. Values range from 0 to 1, and values under 0.5 are not better than a random model [38].
